# Special Issue on Advances in Molecular Simulation

**DOI:** 10.3390/ijms222212484

**Published:** 2021-11-19

**Authors:** Małgorzta Borówko

**Affiliations:** Department of Theoretical Chemistry, Institute of Chemical Sciences, Faculty of Chemistry, Maria Curie-Skłodowska University in Lublin, 20-031 Lublin, Poland; borowko@hektor.umcs.lublin.pl

Rapid advances are taking place in the application of molecular simulations to study complex systems. Molecular simulations are widely used in physics, chemistry, biology, material science, engineering, and even medicine. Each study conducted in a purely theoretical manner can be treated as a simulation. The behavior of a considered system can be modeled using different computational approaches. First, the “usual” theories can be utilized for this purpose. Among them are simple phenomenological theories, advanced quantum methods, and sophisticated statistical–thermodynamic calculations. Second, we can apply various special procedures that allow us to mimic real systems and their evolution over time, namely molecular dynamics, the Monte Carlo method, cellular automata, or genetic algorithms. The most popular simulation techniques of this type are molecular dynamics (MD) and the Monte Carlo method (MC). Impressive progress has been made in the development of different versions of these methods.

A detailed description of molecular interactions is always crucial in simulations. Various force fields are developed and permanently improved to estimate the interaction potentials between atoms or coarse-grained particles. The traditional force field provides a specific function for potential energy and a set of its best-fitted parameters. The potential is fit to the results of quantum mechanical calculations and, usually, to certain experimental measurements. The accuracy of molecular interaction description can be improved by the use of machine learning potentials.

During a typical molecular simulation, the trajectory of the system in the configuration space can be generated stochastically, based on the laws of mechanics, or both. The rules for accepting these states take into account changes in the energy of the system. Simulations can be carried out in different statistical ensembles, including the extended ensembles used in multicanonical simulations or the parallel tempering method. A great variety of enhanced sampling techniques have been successfully implemented in molecular modeling. Numerous methods for the analysis of simulation data have been also proposed, for example, the thermodynamic integration, the weighted histogram method, finite-size scaling analysis, and many others.

The exceptional popularity of molecular dynamics results, among other things, from the availability of many ready-made software packages that provide all major MD codes with a set of accompanying programs performing most steps of the preparation for a simulation. The user-friendly environment allows writing of the simulation scripts without the need for a deep knowledge of the underlying procedures. Nevertheless, extracting reliable and useful information from the simulation always requires a lot of care. The results should be interpreted in light of all available theoretical and experimental data for the system under study.

The ever-increasing power of computers and the development of modern programming methods make molecular simulations a very effective tool for studying more and more complex systems. The main purpose of this Special Issue is to present a wide range of molecular simulation methods and their applications in various fields.

The first section consists of two review articles concerning the methodology of molecular simulations. The survey written by Roy and Kovalenko covers the applications of the statistical mechanics-based three-dimensional reference interaction site model with the Kovalenko–Hirata closure molecular solvation theory in different simulations. They show that this approach can be an essential part of a multiscale modeling framework for the simulation of biochemical systems involving liquid media of complex composition.

Next, Cao and Tian present an excellent overview of the application of “dividing and conquering” and “caching” principles in the development of molecular modeling algorithms. They describe algorithms for the coarse-graining, the enhanced sampling, and the local free energy landscape approach. Differences, connections, and potential interactions among these three algorithmic lines are discussed.

The second part is a collection of original scientific articles devoted to the modeling of different molecular systems. These works can be divided into three groups.

The first series of articles deals with the methodology of theoretical calculations. Mun, Sakai, and Kim propose the quantum mechanical approach for strongly ionizing media. They calculate time evolution operators, employing a time-dependent unitary transformation method within the Kramers–Henneberger framework. The method can be applied to atoms or molecules exposed to strong laser fields. In turn, Shtyrov et al. developed a simplified methodology to study spectral properties of rhodopsins. This approach allows estimation of the direct effect of a charged or polar residue substitution on the first absorption band maxima without extensive quantum-mechanical calculations using only a rhodopsin three-dimensional structure and the data provided in the article. The article written by Lai, Jia, and Chi shows new developments in the open-source Monte Carlo simulation tool gMicroMC (Med. Phys. 2020, 47, 1958). They use this method for the transport simulation of protons and heavy ions and the concurrent transport of radicals in the presence of DNA. Herranz et al. present the simulator-descriptor suite (Simu-D) and use it to model numerous polymer-based systems under extreme conditions of concentration, confinement, and nanofiller content. The simulator involves different MC algorithms, including localized, chain-connectivity-altering, identity-exchange, and cluster moves in various statistical ensembles. The simulator includes various MC algorithms, namely localized changing links of the chain, identity exchange, and cluster movements in statistical teams, while the descriptor identifies similarity of computer-generated configurations with respect to reference crystals in two or three dimensions. It has been shown that the Simu-D can be a useful tool in the studies of entropy- and energy-driven phase transition, adsorption, and self-organization of polymer systems.

The second group of articles covers a broad spectrum of applications of molecular simulations in biomedical research. The article by Ahmad et al. is devoted to the modeling of antibody–antigen interactions. They investigate TLR4 antibody-binding epitope using the computational-driven approach. This computational technique involves the construction of interaction networks, epitope prediction, molecular docking, MD simulations, and binding-free-energy calculations. The method can be a promising tool for the development of new monoclonal antibodies. Choi et al. demonstrate the binding mechanism of selected ligands to the coronavirus 3-chymotrypsin-like-protease through docking and MD simulations. The interactions with SARS-CoV-2 proteases and binding energy were calculated. The study can be helpful for the development of novel and effective, antiviral compounds. The article by Rzecki et al. is on the new computational approach for the comparison of antimicrobial effectiveness among different types of Gemini surfactants. In this study, MD simulations are used to investigate the incorporation of these agents into the model bacteria membrane. The development of new antimicrobials is one of the most serious problems facing medicine today.

The last group of works concerns simulations of various physical phenomena in complex systems under different thermodynamic conditions. Eun presents the MD simulation study of the osmosis-driven water transport through nanochannels. Kobayashi et al. propose a possible mechanism of turbulence suppression in surfactant aqueous solution based on the performed large-scale dissipative particle dynamics simulation. Adsorption on ligand-tethered particles is also investigated using coarse-grained MD simulations (Borówko and Staszewski). Patrykiejew presents the results of MC simulation of the phase behavior of two-dimensional systems of Janus-like particles on a triangular lattice. Moreover, Szeleszczuk et al. investigate the isosymmetric structural phase transition of chlorothiazide using periodic Density Functional Theory calculations and ab initio MD simulations. They prove that DFT calculations enabled the prediction of the transitions of this type. Finally, the structural and dynamical properties of colloids with competing interactions confined in slit pores were investigated using MC and MD simulations (Serna, Góźdź, Noya).

We hope that this Special Issue reflects the power of molecular simulation as an effective research tool in many fields and can provide an impetus for further fruitful studies.

Finally, I wish to express my sincere thanks to each of the authors for their contributions.

